# Recurrent Bilateral Breast Abscesses after Sternotomy

**DOI:** 10.1155/2012/160624

**Published:** 2012-08-09

**Authors:** Hamza Cinar, Ali Naki Ulusoy, Emir Fatih Kaya, Gökhan Lap, Kagan Karabulut, Ayfer Kamalı Polat, Gökhan Selcuk Özbalci

**Affiliations:** Department of General Surgery, Ondokuz Mayıs University, Samsun, Turkey

## Abstract

Median sternotomy is the most commonly used incision in cardiothoracic procedures. Development of breast abscess after sternotomy is a very rare situation. We present a case of sternal wound infection with recurrent bilateral breast abscess after sternotomy. Our case is the first and only case in the literature due to the presence of sternal wound infection with recurrent bilateral breast abscess after sternotomy.

## 1. Introduction

Median sternotomy which is used as a standard incision in cardiothoracic surgery was first described by Milton in 1897, and now it is a routinely used incision in cardiothoracic surgery [1]. The incidence of sternal wound infection ranges between 0.4–5% [2], but sternal wound infection in combination with a breast abscess is an extremely rare complication in the literature.

## 2. Case Report

Aortic valve replacement was performed to a 30-year-old female patient weighing 55 kg with median sternotomy incision due to aortic valve regurgitation. The patient who had no additional disease was discharged at the 6th day postoperatively. On regular examination in the postoperative 14th day, it was seen that sternotomy incision had healed and there was not any focus of infection. After 4 weeks postoperatively, the patient detected a mass under the right breast areola, and the medical examinations revealed a 3 × 2 × 2 cm abscess under the areola at the level of 5–7 o'clock line, and drainage was performed under local anesthesia. Open wound dressings were applied to abscess zone, and oral ampicillin-sulbactam treatment was started and the abscess was completely healed.

During follow-ups, 4 and 6 months after aortic valve replacement surgery, 2 more abscesses formation were developed under right breast areola having the same characteristics. First abscess and foci of abscess were drained under local anesthesia and they were successfully treated by ampicillin-sulbactam therapy. During follow-ups, the patient's physical examination and breast ultrasound revealed a 5 × 3 × 3 cm mass under right breast areola extending to lower quadrants of breast at 3 o'clock to 9 o'clock line and a 2 × 2 × 1 cm foci of abscesses in lower outer quadrant of her left breast between 3 o'clock and 5 o'clock line 15 months later after aortic valve replacement. A thin tract was detected between abscess in right breast with sternotomy incision line. The skin was found to be thinned at sternotomy incision line. The subcutaneous prolene sutures were determined to extend to both breast tissues. An infection was visualized over skin and subcutaneous tissue over sternotomy incision line, but there is no bone infection.

İnfection over sternotomy incision was consulted by cardiovascular surgeon, but no additional suggestions other than antibiotic treatment was done. A surgical intervention was planned for breast abscess. Subcutaneous mastectomy was performed for abscess in right breast due to small-breast tissue and the patient's own request. During the surgery, the fistula tract was detected between abscess in lower quadrant of right breast and sternotomy incision line, and fistula tract was removed during subcutaneous mastectomy. Surgical drainage and segmental mastectomy were performed to left breast abscess lying between 3 o'clock and 5 o'clock line. Also in left breast, the region between abscess zone and sternotomy incision line was excised due to possible fistula tract. Subcutaneous prolene sutures in bilateral breast tissues were removed.

**Figure 1:**
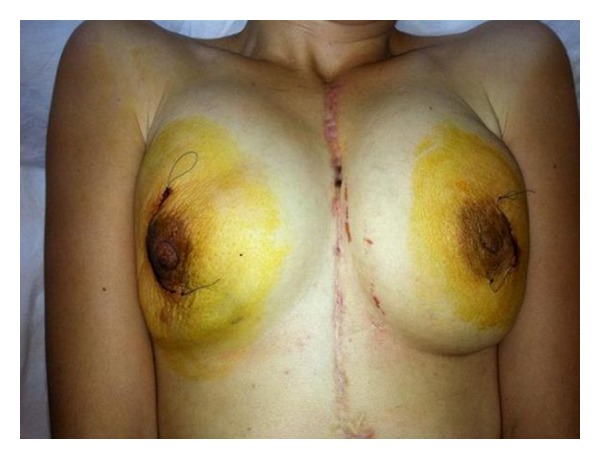
Right subcutaneous mastectomy and left segmental mastectomy were performed to the patient with bilateral recurrent breast abscesses after sternotomy. Photo being taken at postoperative 3rd day.

Pathological examination of the surgical material revealed chronic granulomatous mastitis, and *Staphylococcus aureus* growth was detected in the culture. Ampicillin-sulbactam-treated patient was discharged at postoperative day 3 (Figure 1). During follow-up visit at 3rd month postoperatively, breast lesions were seen to be completely healed.

## 3. Discussion

Today, the median sternotomy is the most commonly used incision in cardiothoracic procedures, and more than 700,000 incisions/year are done only in United States of America (USA) [3].

After median sternotomy, presternal (cellulitis, sinus tracts, and abscesses), sternal (osteomyelitis and separation), and retrosternal (mediastinitis, hematoma, and abscess) complications can be observed. Also breast necrosis case reports were also reported very rarely in the literature [4].

Recurrent subareolar breast abscesses are very rarely seen in nonbreast feeding women and was first described by Zuska et al. in 1951 [5]. Breast abscess after sternotomy was first described by Jansen et al. as a case report in 2002, and it is still the only known case in the literature [6], but our case is the first and only case in the literature due to the presence of sternal wound infection with recurrent bilateral breast abscess after sternotomy.

Although not relatively very common, wound infections after sternotomy are very serious complications. Mortality rates from sternotomy infections were as high as 50% before the 1970s, in recent years, with the use of extensive sternal debridement, muscle and/or omental flaps, vacuum-assisted closure (VAC), and improved antibiotics mortality rates decreased to about 10% [7]. The most common agents that cause sternal wound infections are *Staphylococcus aureus* and *Staphylococcus epidermidis* [8]. In the literature, many risk factors, such as age, gender, obesity, diabetes, chronic obstructive lung disease, and surgical technique, were found which may cause the development of wound infection after sternotomy [9].

The data describing presence of sternal wound infection after sternotomy with breast abscess or stand-alone development of breast abscess is very limited. Only data related to the association of sternal wound infection and breast abscess is the case report which was introduced by Jansen et al. in 2002. In the case presented by Jansen et al., coronary artery bypass was performed to a 62-year-old female patient using median sternotomy. The patient was discharged 5 days after surgery, and it was observed that sternal incision was healed completely. A mass was noticed at left breast upper inner quadrant after 2 weeks postoperatively. The breast ultrasound and fine needle aspiration biopsy of the mass have been identified as an abscess. The patient's abscess was drained under local anesthesia, and abscess zone was left open. Abscess dressings were made for 16 weeks, but no recovery was seen to the desired level. After contrast-enhanced computed tomography and fistulography examinations, a fistula tract between breast abscess and sternum was identified as in our patient. The patient underwent surgery and breast abscess, fistula tract and distal 1/3 portion of sternum were excised. Bilateral pectoralis muscle flaps were prepared and placed at the excised sternum part. Growth of *Staphylococcus aureus* was identified in patient's excised sternum piece and breast abscess material as in our patient. Two months after, breast abscess and sternal flap reconstruction were observed to complete recover [6]. Both in our case, the reason for recurrence of breast abscess despite treatment is that we thought only breast abscess and application of the treatment only for the abscess. During the treatment of breast abscesses that occurred after sternotomy, possible fistula tract between the incision line and the breast tissue may be kept in mind.

## 4. Conclusion

Development of breast abscess after sternotomy is a rare and only seen in the form of case reports. Abscess formation should not be dismissed in patients with breast masses who underwent a sternotomy. The source of abscess may be associated with this sternotomy incision and a multidisciplinary approach should be done.
